# Muscle Function and Cognitive Performance in Autosomal Dominant AlzheimerDisease Insights from the PSEN1‐E280A Colombian Kindred

**DOI:** 10.1002/alz70857_105868

**Published:** 2025-12-25

**Authors:** Elkin Garcia‐Cifuentes, Juan Camilo Becerra‐Mateus, Claudia Aponte, Daniel Vasquez, Paula Ospina, Claudia Muñoz, Yamile Bocanegra, Sonia Do Carmo, David Fernando Aguillón Niño, A. Claudio Cuello, Yakeel T. Quiroz

**Affiliations:** ^1^ Grupo de Neurociencias de Antioquia GNA, Medellin, Antioquia, Colombia; ^2^ Grupo de Neurociencias de Antioquia, Facultad de Medicina, Universidad de Antioquia, Medellín, Antioquia, Colombia; ^3^ Grupo de Neurociencias de Antioquia, Universidad de Antioquia, Medellín, Antioquia, Colombia; ^4^ Grupo de Neurociencias de Antioquia, Facultad de Medicina, Universidad de Antioquia, Medellín, Colombia; ^5^ McGill University, Montreal, QC, Canada; ^6^ Massachusetts General Hospital, Harvard Medical School, Boston, MA, USA; ^7^ Grupo de Neurociencias de Antioquia, University of Antioquia, Colombia, Medellín, Antioquia, Colombia

## Abstract

**Background:**

Carriers of the PSEN1‐E280A variant, the largest known autosomal dominant Alzheimer's disease (ADAD) cohort, typically experience mild cognitive impairment by age 44 and dementia by 49. Emerging evidence suggests gait speed (GS) may be an early marker of cognitive decline. While past research focused on older adults, recent studies explore muscle decline in younger individuals. This study examines the relationship between GS and cognition within the Colombian PSEN1‐E280A cohort.

**Method:**

The study included 134 individuals from families affected by early‐onset ADAD with the PSEN1 gene variant. Among them, 66 were carriers (including 5 with mild cognitive impairment and 5 with dementia), while 68 were non‐carriers. The participants had a mean age of 32 years (SD = 8.72), with 49.3% being male. Cognitively‐unimpaired status was defined by Functional Assessment Staging (FAST) scores <2. Muscle function was assessed through single‐task GS, and its association with cognitive function and functional stages was analyzed. Univariate and multivariate analyses were conducted to explore these relationships.

**Result:**

The carrier group had a mean GS of 1.56 m/s, compared to 1.64 m/s for the non‐carrier group (*p* = 0.307). On the MMSE, all carriers scored an average of 26.55 (SD = 4.56), while non‐carriers scored 28.81 (SD = 1.24), a statistically significant difference (*p* < 0.001). GS was significantly slower in the cognitively‐impaired group compared to cognitively‐unimpaired group (1.37 m/s vs. 1.63 m/s; *p* = 0.042). Pearson correlation analysis revealed a weak positive correlation between GS and MMSE (*r* = 0.18; *p* = 0.041), with this effect being more pronounced in the impaired group.

**Conclusion:**

Our findings show that single‐task GS does not differentiate young individuals with ADAD due to PSEN1‐E280A but is significantly associated with global cognitive function, particularly in cognitively‐impaired individuals. This suggests that gait impairments emerge as cognitive decline progresses, consistent with findings in sporadic Alzheimer's disease, where motor dysfunction often appears in early symptomatic stages. These results reinforce the link between motor decline and cognitive impairment. Further research is needed to explore the interplay between cognitive and physical performance in this population.

